# Solar-powered radio tags reveal patterns of post-fledging site visitation in adult and juvenile Tree Swallows *Tachycineta bicolor*

**DOI:** 10.1371/journal.pone.0206258

**Published:** 2018-11-08

**Authors:** Teresa M. Pegan, David P. Craig, Eric R. Gulson-Castillo, Richard M. Gabrielson, Wayne Bezner Kerr, Robert MacCurdy, Steven P. Powell, David W. Winkler

**Affiliations:** 1 Department of Ecology and Evolutionary Biology, Cornell University, Ithaca, NY, United States of America; 2 Department of Biology, Willamette University, Salem, OR, United States of America; 3 Technology for Animal Biology and Environmental Research (TABER), Cornell University, Ithaca, NY, United States of America; 4 Department of Mechanical Engineering, University of Colorado Boulder, Boulder, CO, United States of America; University of Tulsa, UNITED STATES

## Abstract

The availability of small, lightweight tracking devices enhances our ability to study birds during mobile phases of their lives. Tree Swallows *Tachycineta bicolor*, a model species of wild songbird, are well-studied during their breeding season; but our understanding of their biology at other times of the year, when they are not tied to the fixed location of a nest, is more limited. We developed a lightweight radio tag with no battery (solar nanotag) to study the movements of small animals, and we deployed it to explore the behavior of Tree Swallows after the end of their summer breeding season. We tagged 32 breeding adult swallows and 36 juveniles and monitored their presence and absence at the breeding site during the post-fledging period. Although our observations are based on very small sample sizes, the tags revealed previously unknown patterns in Tree Swallow behavior during the post-breeding season. Some Tree Swallow fledglings continued to visit the site repeatedly in the months following the nesting season, with the latest detection occurring on September 30th; by contrast, all adults had permanently departed by the end of July. These results inform future hypotheses about post-breeding movements in Tree Swallows. But, more generally, the detection of tagged swallows on their distant wintering grounds, seven months after tagging, indicates the potential of studying small passerine movements throughout their entire lifetimes, and suggests a rich array of applications for these “Life Tags” to study the movements of small animals world-wide.

## Introduction

The time between fledging and the onset of migration (or the “post-fledging period”; [1]) is a consequential period in the life of migratory songbirds: juveniles experience high mortality during the post-fledging period [1–3], and they may use this time to prospect for future breeding sites [4,5]. Fledglings may search for areas with high food abundance [6,7], and their movements may help them create targets for navigation back to the area during spring migration [7,8]. Variation in fledgling and juvenile movement behavior may also be correlated with future dispersal decisions: Morton [9] found that juvenile male White-crowned Sparrows (*Zonotrichia leucophrys*) who stayed at natal sites long after fledging were more likely to return to breed there after migration. A better understanding of movement patterns between fledging and territory establishment will lead to critical advances of understanding about dispersal, a highly consequential process for population dynamics and evolution. For example: Are dispersal decisions more strongly affected by the nestling period or the post-fledging period? What factors during the post-fledging period influence dispersal, and how do these vary by context? Although the importance of the post-fledging period is clear, it has historically been difficult to study [1,10]. Recent telemetry studies have greatly added to our understanding of survival patterns and parental care during the post-fledging period [3] and the habitats used by fledglings [7].

We used solar-powered radio tags to investigate patterns of natal site visitation in adult and juvenile Tree Swallows during the post-fledging period. These solar nanotags were produced by the Technology for Animal Biology and Environmental Research (TABER) group at Cornell University, and they have been improved and are now being sold as “Life Tags" by Cellular Tracking Technologies, Cape May, NJ, under license to Cornell University. Because these tags are powered by solar cells and have no batteries, they have no set limit to their lifespan and, at 0.7 grams, they are light enough to be carried by many small passerines. A unique identification code is transmitted by each tag, allowing a large number of individuals to be monitored on a common frequency. The tags transmit their ID code approximately once every 2 seconds during daylight hours, and they are receivable from a range of approximately 2 km with base stations equipped with Yagi antennas.

Tree Swallows are considered a model organism for wild passerines [11], but virtually nothing is known about their post-fledging behavior [12]. Our radio tag data allow us for the first time to describe visitation patterns of Tree Swallows at the breeding site after breeding is finished. We also used our radio tag to investigate whether adult and juvenile Tree Swallows differ in behavior after the breeding season. Juveniles of many passerines remain in breeding areas later in the season than adults (e.g., [13]) and differ in migration phenology (e.g., [14]), so we hypothesized that adults would depart the breeding site before juveniles. While these previous studies use banding data to analyze differences between adults and juveniles over broad spatial and temporal scales, our data allow us to test whether such differences are evident even at the breeding site in the time immediately after breeding is finished.

## Methods

We studied Tree Swallows at the Cornell University Experimental Ponds Facility, Unit 1, in Ithaca, New York, between June and October of 2015. The site hosts a colony of Tree Swallows nesting in man-made nest boxes around a system of ponds. This colony has been monitored regularly each summer for over 30 years. Further details about the site, and the general methods used each summer, are described in [15]. All the work reported here was conducted under an approved Cornell animal welfare protocol (IACUC 2001–0051) to DW Winkler.

### Tagging juveniles

We tagged juvenile Tree Swallows while they were still in the nest. The nestlings used in this study were subjected to an experimental manipulation of the stress hormone corticosterone. This study is described in full in [16], but that study provided no evidence of any large effect of corticosterone on movement patterns that would interfere here with the interpretation of all marked juveniles as showing natural patterns of movement.

We tagged nestlings between 10 and 18 June 2015. Tagged nestlings were between 14 and 17 days old, with 75% tagged on day 15. Nestlings are unable to fly at this age and were returned to their nest boxes after tagging.

**Fig 1:**
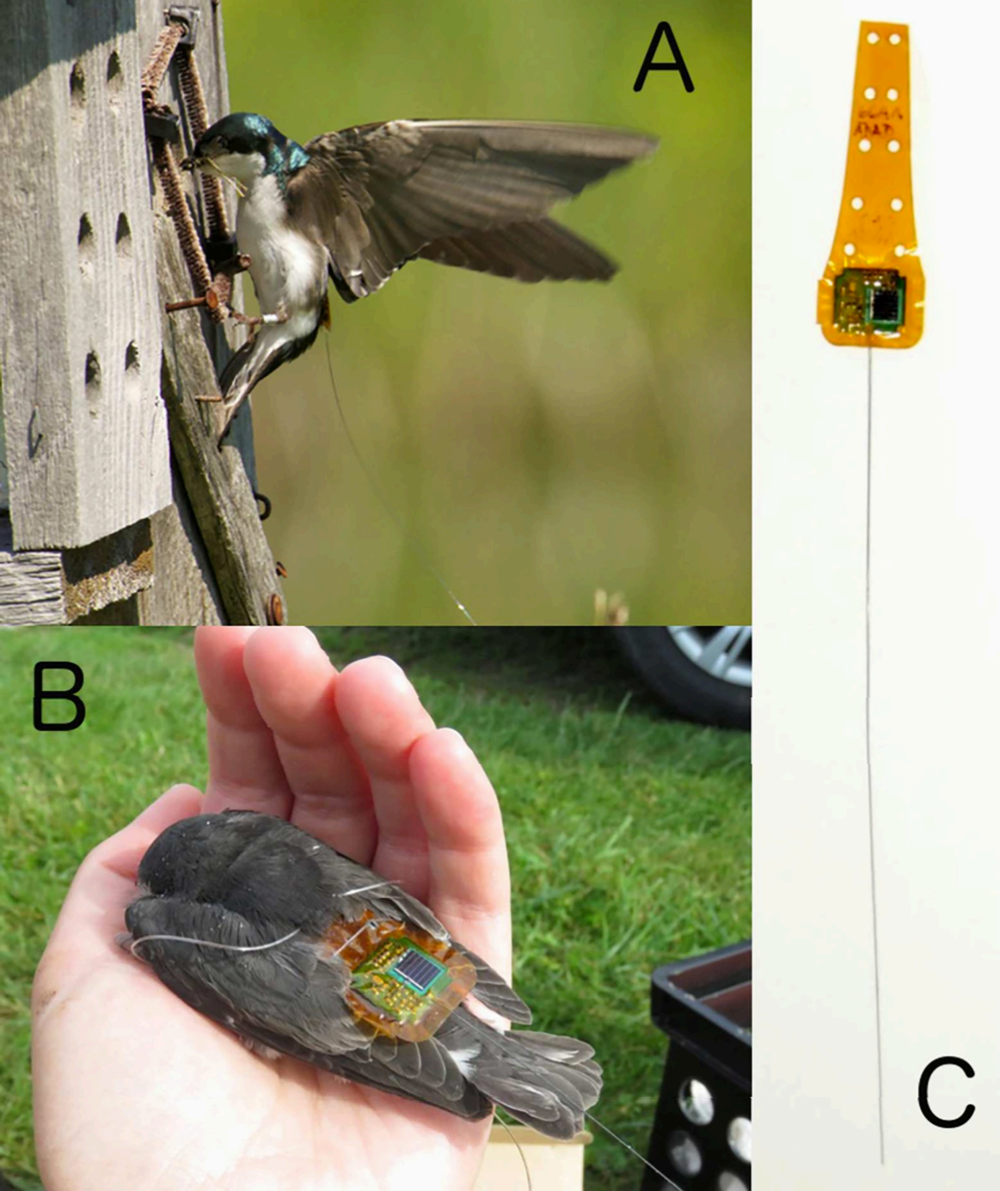
Images of the solar-beeper tag. A) a tagged adult male coming to his nest box with food for his nestlings; B) a nestling just after being tagged, showing how the polymer harness keeps the solar panel from being covered by feathers; the strings visible in the photo were clipped before it was returned to its box; and C) a tag by itself, showing the full polymer sheath and antenna. The solar panel is the black rectangle on the right side of the circuit board. The tongue (here extending toward the top of the photo) of the mylar sheath is designed to be trimmed based on the size of the bird, and in Tree Swallows we trim it so that only the 4 holes closest to the tag are left on the harness. The harness is tied through these holes.

Before tagging, we weighed nestlings and attached tags to the heaviest nestling from each experimental group in each nest; thus, an equal number of corticosterone-manipulated and control individuals were tagged [16]. We tagged the heaviest nestlings to maximize the survival chances of tagged nestlings [17]. We attached tags using a harness [18] made of 0.5-mm Stretch Magic polymer beading twine (Fig 1). Leg loops passed through the tag and the open ends were tied on top of the tag with a square knot secured with a small amount of cyanoacrylate glue. At one nest we tagged a single nestling; at all other nests we tagged two. Ultimately, 36 nestlings fledged with a tag. Five nestlings with tags (~12%) died before fledging. We have no reason to think tagging the nestlings caused elevated rates of mortality; 22% of non-tagged nestlings from these nests that lived to day 12 also died before fledging.

### Molecular analyses

We sexed nestlings using DNA from red blood cells and a standard protocol for sexing Tree Swallows (for details, see [19]). Adults were sexed by plumage and/or presence of a brood patch (which only females possess).

**Fig 2:**
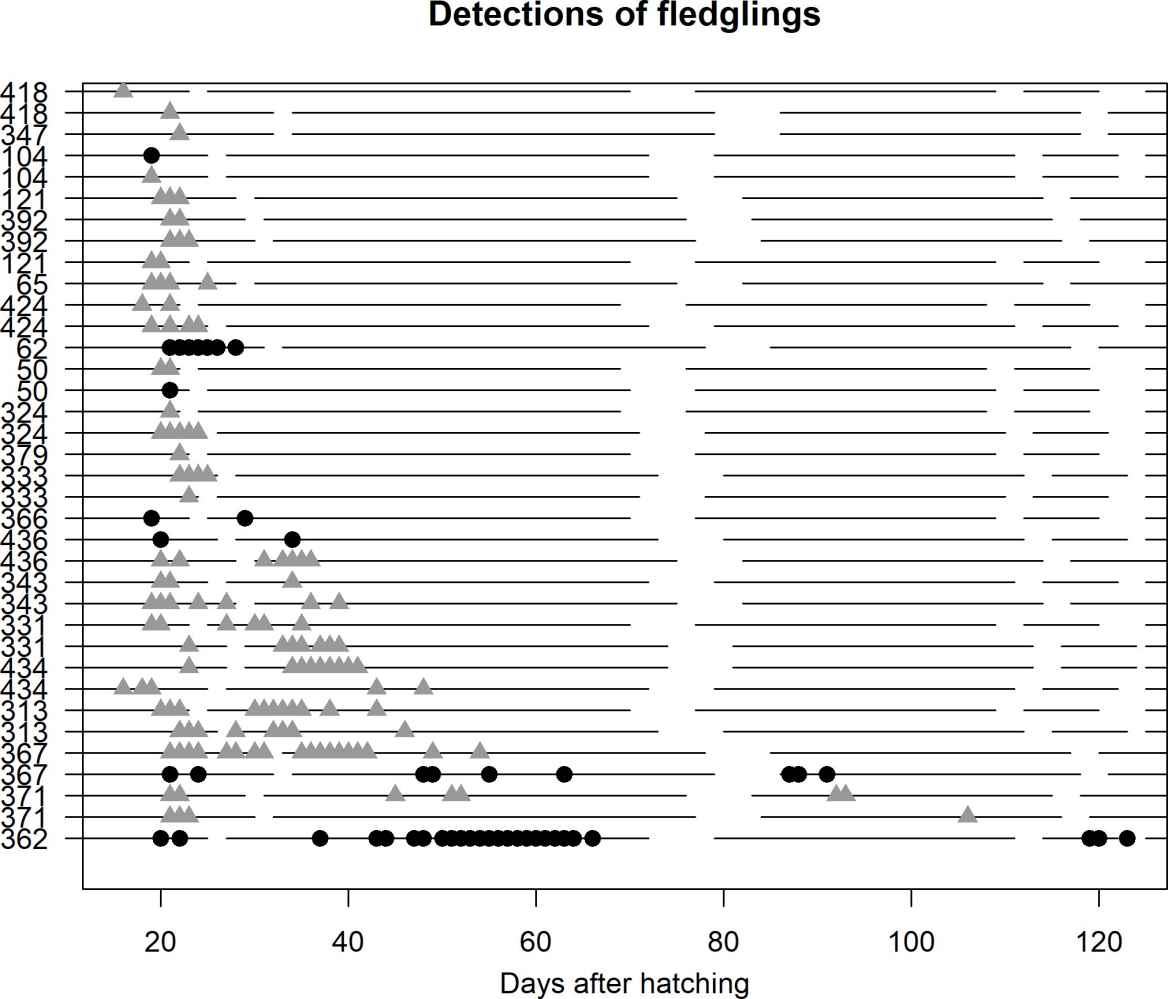
Post-fledging detection pattern for each tagged fledgling. Some juveniles continued to visit the natal site for days or weeks after fledging. Females are shown with black circles and males with gray triangles. Each symbol represents one day on which the individual was detected at the site; the first day on which a symbol appears for a given individual is its fledge day. Individuals are ordered from top to bottom by latest detection date. Breaks in horizontal lines represent days on which the base station was not functioning. The nest number of each fledgling is displayed on the y axis and the x axis is the number of days since a given individual’s natal nest hatched.

Adult male Tree Swallows average heavier than females [20] and, because heavy nestlings were selected for tagging, 29 of 36 tagged fledglings (80.6%) were males (Fig 2).

### Tagging adults

We tagged 32 adults (13 females and 19 males) breeding at the site between 4 June and 14 July 2015. Adults were captured after their eggs hatched, usually when nestlings were 6 days old or older. We tagged adults opportunistically based on availability of tags during the periods when adults were being captured; adults did not come from the same nests where we tagged nestlings, except for one male at a nest with tagged chicks. We weighed adults before tagging to avoid tagging the few individuals in poor condition (those weighing < 17.5 g). Tags with a harness weighed 0.7 g; the 17.5 g cutoff insured that no birds had tags that weighed more than 4% of their body mass.

### Presence/Absence at the breeding site

**Fig 3:**
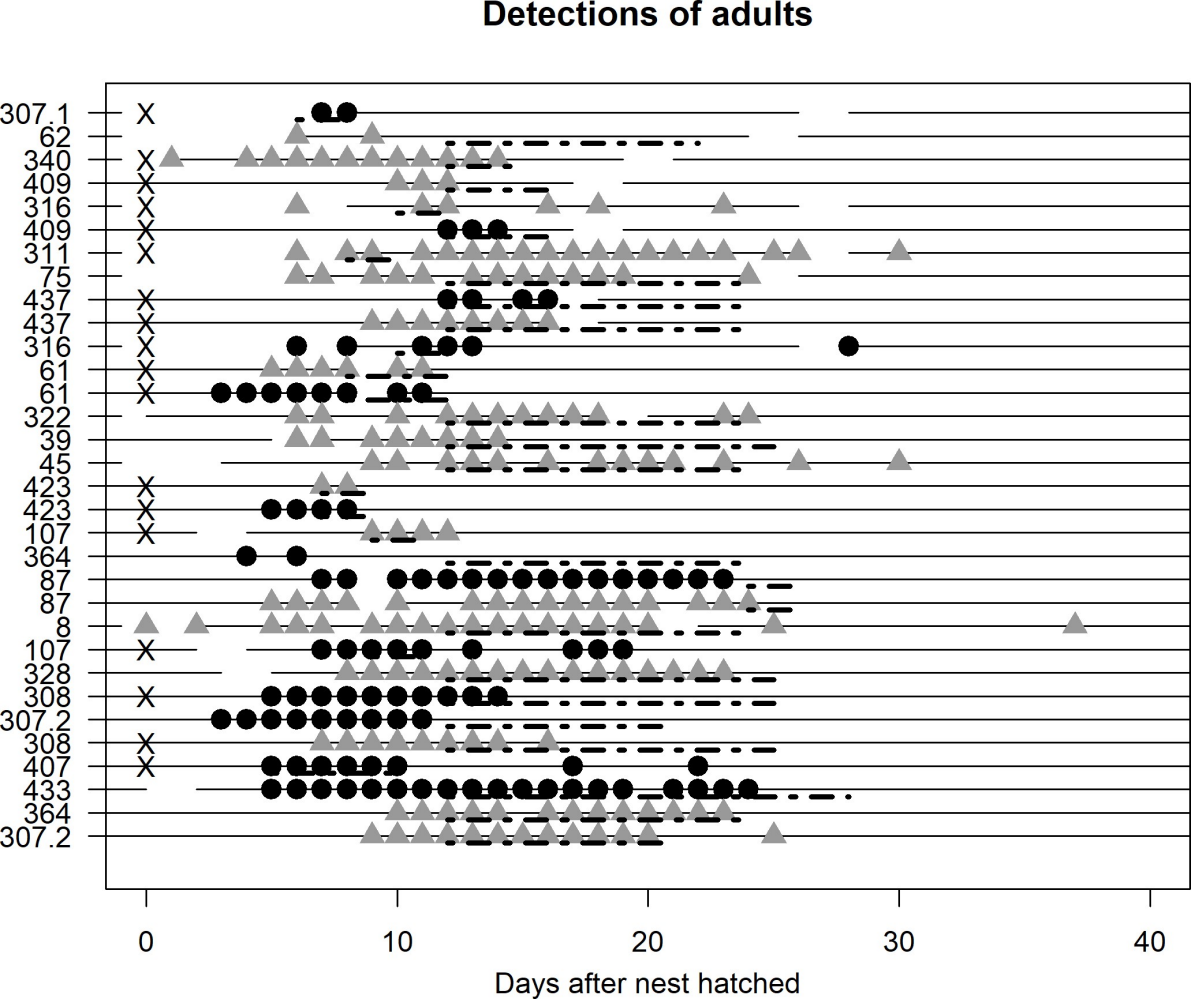
Detection patterns for each tagged adult. The hatch days for each adult’s nest are considered day 0 in this figure, but the nests did not all hatch on the same day. Each symbol represents one day on which the individual was detected at the site; the first appearance of a symbol represents the day that individual was tagged, which in some cases is during a base station off-period. Females are shown with black circles and males with gray triangles. Individuals are ordered from top to bottom by latest detection date. Breaks in horizontal lines represent days on which the base station was not functioning. The nest number of each adult is displayed on the y axis; in some cases we tagged both adults in a pair. Each nest’s fate interval (the period of uncertainty around the nest fate date; see description of “latency to departure” in statistical analyses section) is shown with a dashed line set just below the solid horizontal line, starting on the last known day the nest was active and ending on the day the nest fate was confirmed. Nests which failed to produce fledglings are marked with an “X” on the left side of the plot; all others produced at least one fledgling.

We considered the “breeding site” to be a circle with a radius of 500 m centered over the nest box area. This circle, which also represents the area over which tags could be detected by our autonomous receiver, includes all of the nest boxes (which are all no more than 400 m from the center) and a small amount of surrounding area. We placed an autonomous base station receiver with a 1.67-m Diamond X50NA omnidirectional antenna (Diamond Antenna, San Marcos, CA) near the center of the experimental site on 7 June 2015 and removed it on 25 October 2015. During that time period, the station was not operational for ~18 days (13% of total time) because of battery and mechanical issues. For an additional 23 days (16% of total time), it was either not operational for part of the day or its operational status could not be confirmed (see Figs 2 and 3).

### Technical aspects of the tags and receiver

Details of these tags and how to make them are part of a proprietary manufacturing license with Cellular Tracking Technologies (Cape May, NJ), but, in short, the tags consist of a small integrated circuit chip (hereafter, RF-processor) that transmits a 32-bit digital signal. This signal is interpreted as an 8-digit code by the receiver, with each digit representing 4 bits of the 32-bit signal. The ID was coded and transmitted via amplitude-shift keying [21] whenever a capacitor on the tag had sufficient charge to support transmissions. The RF transmission frequency was ~434 MHz and transmission power was 20 mW. The RF-processor took its digital code from an external electrically erasable programmable read-only memory (EEPROM) chip in the circuit. Each tag could be assigned one of over 2^16^ unique codes. The capacitor in the circuit was charged by solar energy produced by a ~6 x 6-mm custom-cut solar panel, the efficiency of which in low light was bolstered by a boost-converter circuit. The transmission of a tag’s code took 2.5 ms. Limited light affects tags by increasing the interval between transmissions, but does not affect signal strength or duration. Signals were sent over a nitinol whip antenna soldered to the printed circuit board. The completed board (including antenna) was heat-sealed between two sheets of mylar polymer with a protruding tongue. Pairs of pass-through holes were incorporated into the polymer tongue for leg loop attachment points of a harness [18] on the birds (Fig 1), with pairs of holes further up the tongue useful for larger birds with longer feathers. For swallows, we used the bottom two pairs of holes, cutting off the remainder of the tongue on deployment. Signals from tags were received by a custom-made base station with a receiver circuit connected to a proprietary data logger. The receiver recorded the 8-digit code from each signal reception along with a timestamp, and these were written to removable flash memory and retrieved periodically by field crew members.

### Lifespan of the tags

Because they use solar panels rather than batteries, these tags were intended to have a working lifespan of more than one year. However, this first generation of tags had issues with antenna attachment because, to save mass, we used only solder to attach the antenna to the circuit board, believing that the mylar sheath would protect the attachment point; however, the adhesive between mylar layers near the attachment point was eroded by antenna movement over time, allowing strain on the antenna attachment that caused it to fail. This problem was remedied in subsequent versions of the tag by a reduction in antenna mass and stiffness, structural reinforcement of the antenna-board connection and a vibration damping connection between the mylar sandwich and antenna base.

### Searching for tagged Tree Swallows beyond the study area

After fall migration had begun, we searched for tagged Tree Swallows in the field on migratory staging and wintering grounds. On 17 & 26 July, and 23 & 28 August 2015, TMP and ERGC brought receivers equipped with Yagi antennas (Diamond Antenna, San Marcos, CA) to Montezuma Wildlife Refuge, ~60 km north of the breeding site. Tree Swallows stage at this site as they prepare for migration, often forming large roosts [22]. On 18–19 September 2015, TMP and DWW, and an undergraduate assistant visited Salem and Cape May, New Jersey, locations of migratory Tree Swallow roosts which are ~330 km and 420 km, respectively, from the breeding site. Between 14 and 19 January 2016, TMP, ERGC, DWW, and DPC took two Yagi-equipped receivers to central Florida (~1700 km from the breeding site), where previous data suggested Ithaca birds spend the winter [23]. We used radar to identify areas where large numbers of Tree Swallows were present [24] and visited roosts and promising habitat to scan for tags. We visited roost areas in Tarpon Springs, Sarasota, and Cape Canaveral, and kept receivers on with antennas deployed throughout our travels in daylight hours to and around these sites.

### Statistical analyses

Data from the base station included a received tag’s code and the date and time the code was received. Data were offloaded from the base station, processed in TextWrangler to remove unusual characters or corrupted reads, and analyzed in R 3.4.1 [25].

Most of our analyses focused on whether a tag code was received on a given day. A tag was considered present at the breeding site on a day if its code was received at least once on that day, and unless otherwise noted, we made no analyses distinguishing the number of times a code was received. We used Wilcoxon rank-sum tests to compare mean values between the adults and fledglings because the data were not normally distributed. We compared the following variables:

#### Latency to departure

Fledgling latency to departure is the number of days between fledging and the first day on which no tag signal from that individual was received. For adults, we used the bird’s nest- fate day (either fledge day or death day) as the benchmark from which to measure latency to depart. However, we usually do not accurately measure fate dates at this breeding colony to avoid disturbing nestlings who are old enough to be at risk of premature fledging. For each nest, there is thus an interval of time between the last confirmed date the nest was active, and the date on which the fate of the nest was first discovered; we refer to this interval as the “fate interval” (Fig 3). We measured latency to departure in adults from the midpoint of their nests’ fate intervals as a proxy for the nests’ fate dates. Some adults first departed the site before the midpoint of their fate interval, giving them a negative value in latency to departure.

#### Number of returns

Number of returns for fledglings was the number of times (between June and October 2015) they returned to the site after being absent for one day or more. Number of returns for adults is similar; we only counted returns that occurred after the fate interval midpoint.

#### Days present post nest

For fledglings, this value represents the total number of days a fledgling was present at the site after fledging (including visits that occurred after periods of absence, but excluding the days they were absent). For adults, this similarly represented the total number of days an adult was present after its fate interval midpoint.

#### Latest detection

For fledglings, latest detection is measured as age in days (rather than days since fledging). For adults, latest detection is measured as the number of days since eggs hatched; thus, these two measures are directly equivalent between adults and fledglings, because both start counting at the hatch date at a given nest.

#### Lighting conditions

We also estimated the lighting conditions under which the tags transmitted normally using light level information from PVEducation.org (http://www.pveducation.org/pvcdrom/calculation-of-solar-insolation).

## Results

### General description of post-fledging behavior

In 7 of 15 cases where both siblings fledged, they fledged on the same day. Most other pairs of siblings fledged within three days of each other (mean difference = 1.2 days). In one nest, a single tagged nestling was found alive in a box six days after its siblings fledged (we placed this nestling on top of the box to encourage it to fledge, and it flew away); at another nest, the fledge date of one sibling is unknown because the nest box was empty several days before that bird’s tag code appeared in the data collected by the receiver.

Fledglings generally first departed the breeding site (i.e., were absent for an entire day) within a few days of fledging, but there were some exceptions (Fig 2). For 11 of 15 pairs of siblings, both left on either the same day or within one day of each other (mean difference = 1.4 days).

Eighteen fledglings visited the breeding site after their initial departure. Most visits lasted only a day or a few days, although some visits lasted more than a week (Fig 2). Although siblings were often present at the site on the same days immediately following fledging, we recorded only two instances of siblings being present at the site on the same day after both were absent, and in both cases they had arrived on different days (but overlapped subsequently). The latest detection of any swallow at the breeding site was a female fledgling on 30 September 2015, 102 days after she fledged.

### Differences between fledglings and adults

Some adults left the breeding site for long periods within a day of being tagged, whereas others stayed until after their nestlings fledged (Fig 3). Though we do not have precise nest fate dates, the tag data suggest that the adults generally depart the breeding site shortly after their nest is finished (whether it fledged or failed) and generally do not return again. In one exception, a female (nest 316, Fig 3) returned for one day 17 days after her nest failed, when other birds were still breeding (unlike some fledglings, which returned after most swallows had left the site). The latest detection of an adult at the breeding site was on 31 July 2015 (the male at nest 307.2, Fig 3), ~5–10 days after his nest’s fledge date.

**Fig 4:**
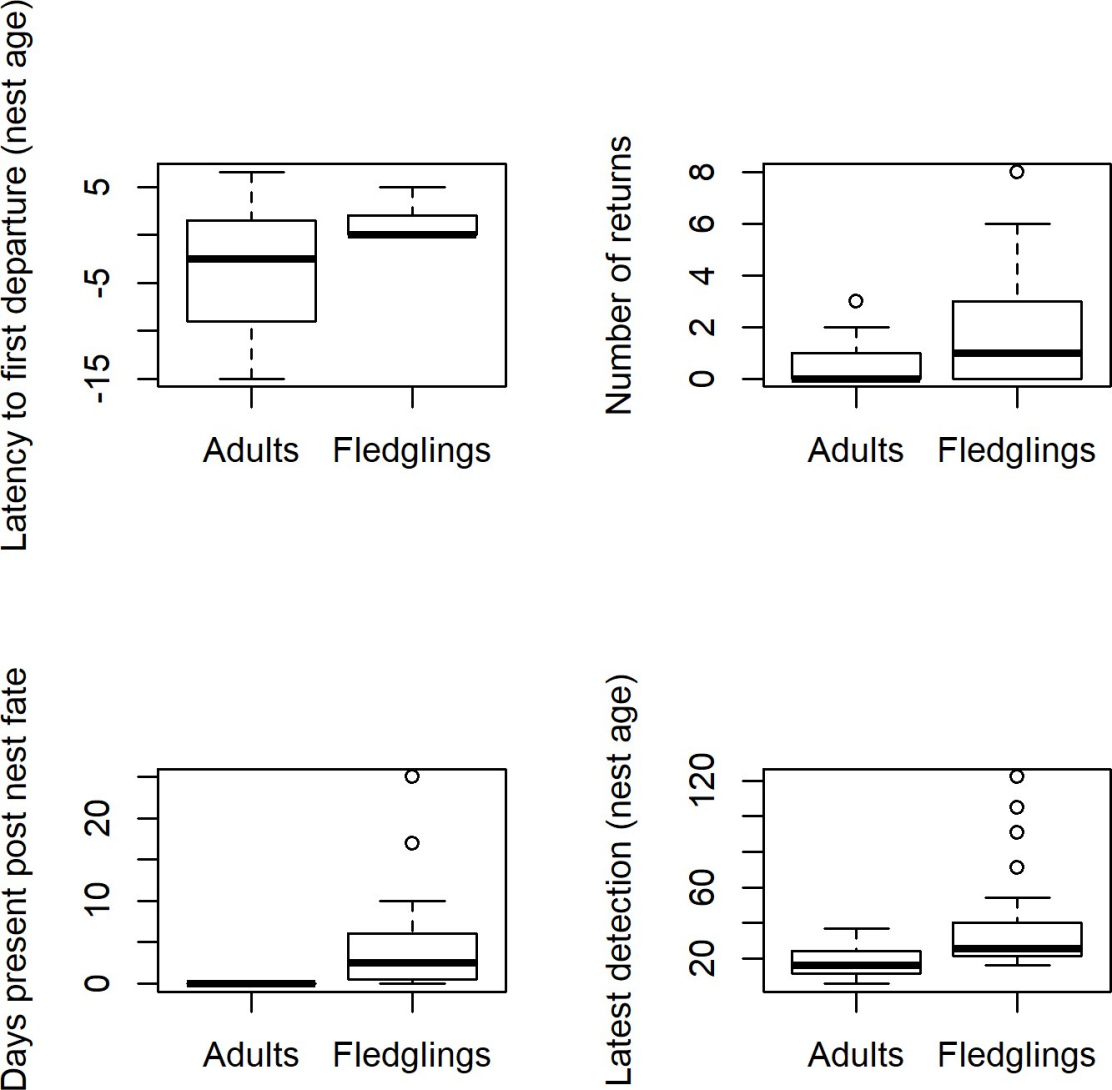
Variation in adult and juvenile post-fledging detection patterns at the breeding site. Boxplots for adults and fledglings are similar except that many adults first departed before their estimated nest fate (which is impossible for fledglings), and the mean latest detection of fledglings is later than that of adults. In all cases, the sample size is 32 adults and 36 fledglings. For latest detection and latency to departure (top left and lower right), the y-axis unit is given as “nest age,” which is the number of days since the hatch date of a given fledgling or of the nest of a given adult.

Adults (N = 32) and fledglings (N = 36) differed in latency to departure because many adults first departed before their nests' fate-dates (Fig 4; Wilcoxon rank sum W = 822.5, *P* = 0.0019). They were similar in number of returns (Fig 4; Wilcoxon rank sum W = 709, *P* = 0.076), but fledglings spent more time at the site after the breeding season and had later mean latest detection dates (Fig 4; Wilcoxon rank sum, W = 144, 918.5 respectively; *P* < 0.0001 for both tests).

### Effects of light level on tag transmission

The tags transmit steadily under lab conditions when illuminated at the equivalent of 7,000 lux (0.055 kW/m^2^). Assuming a clear day, illumination at solar noon at our field site is roughly 110,000 lux (0.869 kW/m^2^) and illumination drops below 7,000 lux within 0.5 hr of sunrise or sunset; this indicates that tag performance may be suboptimal in the 30 minutes after sunrise and before sunset. The tags performed as expected, with tag receptions spread broadly over the majority of daylight hours.

### Encounters with tagged Tree Swallows after the post-breeding season and beyond the main study area

We successfully located tagged Tree Swallows away from the breeding site in New York in July 2015 and in Florida in January 2016 (Table 1). Two fledglings (~6% of total tagged fledglings) and one adult were detected in New York, and three different adults were detected in Florida (~9% of tagged adults). We also received signals from five different tagged Tree Swallows at the Ithaca study area the following breeding season in March and April of 2016 (approximately 10 months after deployment), when swallows were beginning to return from spring migration. One of these returning tagged birds was a nestling in the 2015 season, but it was not detected as a breeder in 2016, nor were any tagged fledglings from 2015 encountered in 2017. In any event, by May, 2016, we began to catch returning adult Tree Swallows with tags missing their antennas. Ultimately, we caught 8 adult birds (5 males, 3 females) in 2016 with broken tags (~22% of the adults tagged in 2015), and no birds were captured that had lost their tag. The tag belonging to one of these birds had been successfully transmitting in early May. We caught no birds at our site in 2016 with functioning tags. As a guide to interpreting return rates for tagged birds, 34 (43%) of 80 untagged females breeding at the study site in 2015 returned to breed in 2016, and 3 (23%) of 13 tagged females returned in 2016. Of these 3 returning tagged females, 2 were captured on their nests in boxes, and both produced viable eggs with hatching chicks, one fledging her brood and the other losing it in a cold snap. And, as a guide to interpreting.

**Table 1:** Locations of Tree Swallow detections away from the breeding site.

Location	Km from breeding site (approx.)	Dates	# tagged swallows detected/approx. # swallows present	Notes
Montezuma NWR, NY	60	17 Jul 2015	3/700	Detected 2 male fledglings and 1 male adult
Montezuma NWR, NY	60	26 Jul 2015	1/100	Detected the same adult as on 17 July
Montezuma NWR, NY	60	23 Aug 2015	0/100	
Montezuma NWR, NY	60	28 Aug 2015	0/100	
Salem, NJ	330	18 Sep 2015	0/100,000	
Cape May, NJ	420	19 Sep 2015	0/100	
Tarpon Spring, FL	1700	14 Jan 2016	0/100,000	
Tarpon Spring, FL	1700	16 Jan 2016	0/100,000	
Sarasota, FL	1700	15 Jan 2016	0/100,000	
Cape Canaveral, FL	1700	17 Jan 2016	3/50,000	2 males and 1 female, tagged as adults. Different individuals than those detected at Montezuma
Cape Canaveral, FL	1700	18 Jan 2016	0/1000	
Cape Canaveral, FL	1700	19 Jan 2016	0/1000	

All the broken tags recovered in 2016 were intact except for the missing antenna, and inspection of the area of antenna breakage indicates that in the normal course of bird activity the antenna moved, first weakening the bond between the sealed layers of mylar protecting its base, and eventually attaining sufficient freedom of movement that it over-strained the soldered junction to the board. The pattern of failure that we observed in spring 2016, where at least five tags were transmitting successfully in April 2016, but no working tags were present at the site by June 2016, leads us to suspect that the antennas were falling off with the re-initiation of the breeding season, and we suspect that entering and exiting nest-boxes, with the inevitable sharp bends and twists to the antenna that this necessarily entails, may have dealt the antennas the final blow. All of this taken together suggests that the antenna failures of these tags were concentrated long after the post-breeding season and that any biasing effect on our data from the 2015 post-fledging period is negligible.

## Discussion

Data from Tree Swallows carrying long-lasting solar radio tags revealed previously unknown patterns in the movement biology of this model species. Unlike some other songbirds [10], adult Tree Swallows move away from breeding areas between the end of their nesting attempt and the onset of migration, gathering in large roosts [21]; but we found that most adults depart the site even before roost formation begins. In central New York, large Tree Swallow roosts tend to begin appearing at the end of July [26], but the mean latest detection date of adults in our study was July 4th; their whereabouts between the end of breeding and the onset of roost formation are unknown. We found one adult swallow and two juveniles in mid-July at a large wetland complex 60 km north of the breeding site, where roosts form later in the summer.

Only fledglings, not adults, returned to the breeding site after July. These fledglings may have returned to the site during the process of prospecting for their future breeding location: in the Purple Martin (*Progne subis*, another species of swallow), juveniles are sometimes known to defend nest cavities at their natal site during the post-fledging period [27]. Across many bird species, individuals that engage in prospecting behavior tend to be those more likely to disperse (e.g., juveniles and failed breeders [5]), so it is unsurprising that young birds returned later in the season than adults: Adult Tree Swallows (including females) are strongly philopatric after their first breeding season [28], while most young Tree Swallows disperse from their natal site instead of recruiting there [29]. Swallow fledglings of other species are known to stay together for up to 12 days after fledging [30–32]. In our study, sibling fledglings were generally present on the same days shortly after fledging (Fig 2), but we did not find that they returned together when visiting the site after their initial departures.

Radio-tagging always presents some risk for birds [33], and there are potential negative consequences of radio tagging for both adult and juvenile Tree Swallows. To mitigate negative effects of tags on nestlings, we recommend that nestling Tree Swallows not be tagged until at least 15 days post-hatching so that, when tagged, they are as large as they will get [17]. We also suggest that no more than two nestlings per nest should be tagged with tracking devices that have whip antennas, to reduce the likelihood that antennas will become twisted together, causing entanglement. We did not find that radio tags reduced the likelihood that nestlings would fledge, but the tags did affect return rate. In our system, about 6–8% of fledglings tend to recruit to the breeding site each year (pers. obs). This means that we would have expected 2–3 tagged fledglings to recruit to the breeding site, even though many young Tree Swallows disperse to different sites to breed [29]; and we mostly tagged males, which are the more-philopatric sex. Even though one tagged nestling returned from 2015, none of the tagged nestlings were found breeding in 2016. Though our sample sizes are very small, we found that radio-tagging had a statistically significant negative effect on return probability of nestlings involved in our corticosterone manipulation, which includes the tagged juveniles discussed here [16]. However, because these first-generation tags lasted less than one year, we cannot distinguish whether tagging affected survival, dispersal, or both.

Tagging adults may also make them less likely to produce fledglings; many nests at which we tagged adults failed to fledge young (see Fig 3). However, we did not conduct a statistical test to see whether this pattern is significant, because we do not have a large enough sample size to control for confounding factors such as periods of cold weather, which are a major cause of nest mortality at our site [34]. The viable chicks produced by the two returning tagged females at least indicate that the tags do not prevent effective copulation and parental care. One of the challenges of producing a tag that can last the lifetime of a bird, is that the tag needs to be attached with its solar panel unobscured by feathers in a way that does not impact the bird negatively over its entire lifetime. Such refinement is the subject of ongoing research.

The results presented here will serve as a starting point for future investigation. For example, actively searching for adult Tree Swallows between their departure from the breeding grounds and the onset of pre-migration roost formation may reveal where they go during this time period, lending insight into the habitats and resources they use as they begin to molt (which Tree Swallows do before migrating) and prepare for migration. Gathering information of this kind is increasingly important because Tree Swallows, like many other aerial insectivores, have experienced population declines in recent years (e.g., [35]). Constructing an array of autonomous receivers in suitable Tree Swallow habitat in the wider area beyond a particular colony would allow for a more detailed understanding of both adult and juvenile Tree Swallow movements. Our successful detection of multiple tagged Tree Swallows at a large wintering roost in Florida demonstrates the potential of using these long-lasting radio tags to increase our understanding of individual behavior outside of the breeding season including migratory biology and habitat use. The fact that all the tagged individuals that we detected in Florida were in the same large flock suggests that some individuals from our study site may stay together throughout the entire year, which may have interesting implications for social behaviors such as mate choice (cf. [36]).

Because the lifespan of these tags is not limited by the weight constraints of a battery, their theoretical lifespan is longer than that of the average passerine. The first-generation version of these tags lasted less than a year because of a weak attachment between the antenna and the tag, but we are confident that current improved versions of the tag will last far longer. This means that these tags can be used to find individuals which make natal or breeding dispersals away from the areas where they were captured. The ability to track individual songbirds for more than a year will facilitate attempts to experimentally manipulate dispersal distance, improving our understanding of this critical yet difficult-to-study event in avian life history.
